# Challenges, Unmet Needs, and Future Directions for Nanocrystals in Dermal Drug Delivery

**DOI:** 10.3390/molecules30153308

**Published:** 2025-08-07

**Authors:** Muzn Alkhaldi, Cornelia M. Keck

**Affiliations:** Department of Pharmaceutics and Biopharmaceutics, Marburg University, Robert-Koch-Str. 4, 35037 Marburg, Germany; muzn.alkhaldi@pharmazie.uni-marburg.de

**Keywords:** nanocrystals, plantCrystals, hair follicle targeting, dermal drug delivery, solubility, high-pressure homogenization

## Abstract

Nanocrystals, defined as crystalline particles with dimensions in the nanometer range (<1000 nm), exhibit unique properties that enhance the efficacy of poorly soluble active compounds. This review explores the fundamental aspects of nanocrystals, including their characteristics and various preparation methods, while addressing critical factors that influence their stability and incorporation into final products. A key focus of the review is the advantages offered by nanocrystals in dermal applications. It also highlights their ability to enhance passive diffusion into the skin and facilitate penetration via particle-assisted dermal penetration. Additionally, the review discusses their capacity to penetrate into hair follicles, enabling targeted drug delivery, and their synergistic potential when combined with microneedles, which further enhance the dermal absorption of active compounds. The review also addresses several commercial products that successfully employ nanocrystal technology, showcasing its practical applications. Summary: Nanocrystals with their special properties are an emerging trend for dermal applications, particularly the development of plantCrystals—natural nanocrystals sourced from plant materials—which represent a promising path for future research and formulation strategies. These advancements could lead to more sustainable and effective dermal products.

## 1. Introduction

Dermal applications encompass a wide range of products designed for topical use, targeting the skin for therapeutic or cosmetic purposes. In the pharmaceutical sector, dermal formulations, such as creams, gels, ointments, and patches, are developed to deliver active ingredients directly to the affected area, providing localized treatment while minimizing systemic side effects [1,2,3,4,5,6,7,8].

The cosmetic industry relies heavily on dermal applications to address aesthetic concerns, supporting the maintenance and improvement of skin health. Products such as moisturizers, anti-aging creams, sunscreens, and serums are formulated to enhance the appearance and texture of the skin, offering benefits ranging from hydration to protection against environmental damage. With the growing consumer demand for effective and innovative skincare solutions, the integration of advanced technologies, including nanotechnology, has become increasingly prevalent [9,10,11,12,13,14].

Consequently, many different approaches (and combinations thereof) that improve skin conditions and/or enhance the dermal penetration of active compounds are available today. This includes, for example, modern types of vehicles such as emulgels [15,16,17] and suspoemulsions [18], the use of skin-friendly chemical enhancers that can temporarily modify the fluidity of the skin barrier (for example, alcohols [19,20,21,22] or phospholipids [23,24,25], or formulations like hydrogels [26,27,28]), and transdermal patches [29,30,31] that aid in drug retention and controlled release for small, lipophilic drugs. Nanocarrier systems such as liposomes [32,33,34], solid lipid nanoparticles [35,36,37,38], cyclodextrins [24,39,40,41,42] and nanoemulsions [43,44,45] are also well-established strategies to improve drug penetration and stability. Additionally, emerging technologies such as stimuli-responsive systems [46,47,48], dissolving microneedles [49,50,51], and 3D-printed devices [52,53,54] are expanding the possibilities for targeted and efficient dermal drug delivery. Moreover, physical methods such as iontophoresis, electroporation, sonophoresis, microneedles, and laser-assisted techniques that physically breach or alter the skin to enhance permeability are also used to improve or modify the uptake of active compounds into the skin [55,56,57,58,59,60,61,62,63,64,65].

**Figure 1:**
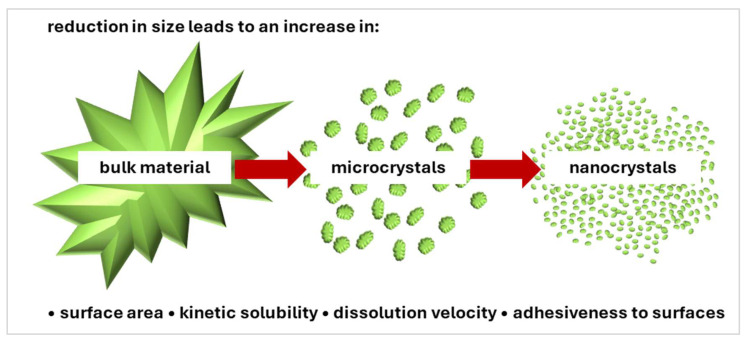
Comparison of physico-chemical properties of nanocrystals in comparison to bulk material and microcrystals.

Besides these above-mentioned strategies, nanocrystals, with their unique physicochemical properties (Figure 1), have emerged as a promising approach in both pharmaceutical and cosmetic formulations. By reducing particle size, nanocrystals can significantly enhance the solubility and permeability of active ingredients, facilitating deeper penetration into the skin layers. This capability improves drug delivery efficiency and allows for the formulation of products with lower concentrations of active ingredients [66,67,68,69,70,71,72,73,74].

As dermatology and cosmetic science continue to evolve, the application of nanocrystals presents exciting opportunities for developing more effective, targeted, and user-friendly dermal products. Understanding the mechanisms by which nanocrystals operate can provide valuable insights into their potential to optimize dermal drug delivery and cosmetic effectiveness [69,70,72,75,76].

The objectives of this review are to explore the fundamentals of nanocrystals, providing a comprehensive overview of their properties, preparation methods, and mechanisms of action as they relate to dermal applications. We aim to evaluate the current applications of nanocrystals in pharmaceuticals, focusing on their role in enhancing the solubility and bioavailability of poorly water-soluble active compounds in topical products. Additionally, this review explores the impact of nanocrystals on cosmetic formulations, investigating their ability to improve dermal penetration of active compounds and overall product performance.

## 2. Fundamentals of Nanocrystals

### 2.1. Definition and Characteristics

**Figure 2:**
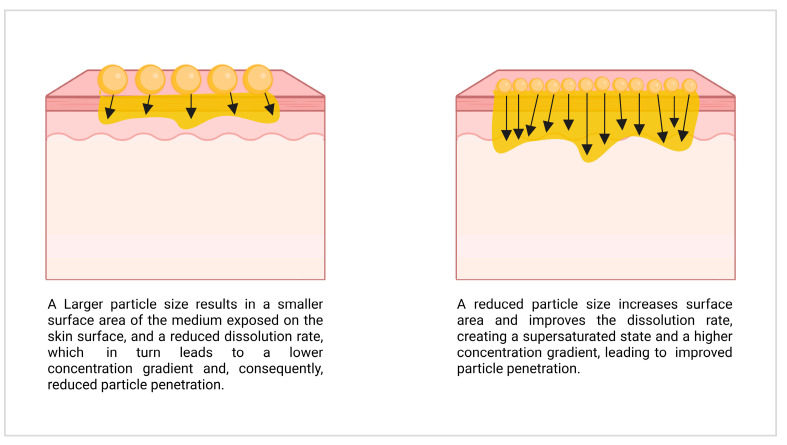
Illustration of the supersaturated state influence on particle penetration.

Drug nanocrystals are submicron-sized crystalline particles of active pharmaceutical ingredients (APIs), typically less than 1000 nanometers in size. They are composed of 100% active material and are primarily produced to enhance the solubility and bioavailability of poorly water-soluble drugs (e.g., BCS class II and IV drugs). By reducing the particle size, the total surface area increases, resulting in a much larger surface area-to-volume ratio compared to their larger counterparts [70,72,76,77,78,79,80,81,82,83,84,85]. Based on the Noyes–Whitney equation, the larger surface area enhances the dissolution rate as in Equation (1).

(1) $$\frac{dC}{dt}=\frac{DS\;(Cs-C)}{Vh}$$

where *dC*/*dt* is the rate of dissolution, *D* is the diffusion coefficient, *S* is the surface area of the solid, *Cs* − *C* is the concentration gradient, *V* is the volume of the medium, and ℎ is the thickness of the diffusion layer [8,84,86]. In addition, a decrease in particle size increases the curvature of the particle surface. According to the Kelvin equation [87], this increases the kinetic solubility of the active compound and creates a supersaturated state. The higher amount of dissolved active ingredient in the supersaturated state leads to a higher concentration gradient and thus enhances the uptake of the active ingredient if passive diffusion is the mechanism of drug uptake [1,8,70,77,87,88] (Figure 2).

### 2.2. Preparation Methods

Nanocrystals for improved solubility of poorly water-soluble compounds were first produced in the early 1990s by wet milling [89]. To date, this remains the most commonly used method for the production of nanocrystals. Later, other techniques such as high-pressure homogenization, precipitation, solvent evaporation, or combinations of these methods were also used to produce nanocrystals. Each method has its benefits and limitations. Therefore, the most appropriate method for the production of nanocrystals always depends on the type and the properties of the raw bulk material, the intended final particle size of the nanocrystals (<100 nm, 100–300 nm, 300–800 nm), and the desired concentration of the nanocrystals in the product (<5%, >10%). In general, smaller-sized nanocrystals with sizes below 400 nm are mainly obtained with bead milling or combinations of the different techniques. High-pressure homogenization or high-speed stirring are the easiest methods and typically form slightly larger particles, which can be beneficial for some applications such as hair follicle targeting [69,70,90,91,92,93].

If the particle size of the nanocrystals is not essential for the overall product performance of the final product, the selection of the production technique can focus on the costs of production, e.g., required equipment, time needed for production, GMP requirements, cleaning procedures, etc. Bead milling—in comparison to high-pressure homogenization—is a low-energy process and is therefore suitable for milling thermosensitive materials, such as antioxidants. However, the milling time is long and can require several days. The separation of the beads from the milled material is also tedious. Nevertheless, the process is well established, and bead mills suitable for use in GMP environments are available. High-pressure homogenization does not have the limitation of bead milling. The production is fast (typically less than 1 h), and the cleaning process is also quick. GMP-compliant high-pressure homogenizers are available for GMP production. The drawback of high-pressure homogenization is the high energy input, which can harm thermosensitive compounds. The combination of both methods has been proven to be optimal for the fast production of nanocrystals for dermal applications [8,67,83,84,94,95,96,97,98,99,100].

### 2.3. Formulation Aspects, Physical Stability, and Transfer into Final Formulations

Nanocrystals are produced via wet milling methods, leading to their dispersion in a liquid. The resulting nanosuspensions are highly dispersed systems, which increases their susceptibility to aggregation due to high surface energy, necessitating the use of stabilizers or surfactants to prevent this aggregation [69,94,101,102,103]. Stabilization can be achieved by using ionic or non-ionic surfactants. Ionic surfactants provide electrostatic stabilization, and hence, nanocrystal formulations of this kind should possess a high zeta potential. However, ionic surfactants can easily interact with the skin and are often considered to be non-skin-friendly. Therefore, nanocrystals for dermal applications should preferably be stabilized with skin-friendly stabilizers. Non-ionic stabilizers are often referred to be skin-friendly. Accordingly, they are recommended to be used for the production of nanocrystals for dermal applications. Non-ionic stabilizers provide steric stabilization, which means they are intended to “coat” the nanocrystals with a thick layer of stabilizer, which then results in a low zeta potential, most preferentially being close to zero mV [1,8,68,70,73,78].

The increased kinetic solubility of the nanocrystals creates a supersaturated environment. This means nanocrystals are thermodynamically highly unstable, and during storage there is always a risk of precipitation of the dissolved active ingredient [1,68]. For this reason, to fully exploit the potential of nanocrystals, formulations that not only allow for a fast and high dissolution of the active ingredient but also allow for a sufficient maintenance of the supersaturated state are needed. Good physical stability and a long maintenance of the supersaturated state can be achieved by producing nanosuspensions with a narrow particle size distribution and by adding protective colloids to the nanocrystal formulation. The narrow size distribution avoids Ostwald ripening, and the addition of protective colloids prevents recrystallization of the dissolved molecules [68,70,88,91,104]. Drying the nanocrystals, for example, via lyophilization, is another option to increase the physical long-term stability of nanocrystals [88,105]. If supersaturation cannot be maintained, large particles without the special properties of the nanocrystals are formed during the precipitation process, which causes the loss of the functionality of the nanocrystals. The development of small-sized and physically stable nanocrystal formulations can be challenging and needs long-time experience not only in the production but also in the characterization of the formulations. This is especially relevant when nanocrystals are incorporated into semi-solid formulations that contain other ingredients that, for example, can change the solubility of the material from which the nanocrystals are made and/or the zeta potential of the nanocrystals, which causes changes in the stabilization mechanism that prevents agglomeration of the particles. Thus, the most stable formulations are obtained with simple vehicles such as hydrogels, with no other ingredients [68,69].

### 2.4. Fate of Nanocrystals After Dermal Penetration

The fate of nanocrystals after dermal application is not yet fully understood, and more research is needed to understand the complex interplay between dissolved molecules, nanocrystals, the vehicle, excipients and/or other ingredients, and the skin. This section briefly summarizes the current knowledge of the fate of nanocrystals after dermal application. Nanocrystals are particles composed of the active ingredient, which can dissolve or partially dissolve in the formulation vehicle. Intact skin with a non-impaired skin barrier can be considered to be a tight barrier in which nanoparticles with sizes >30 nm cannot penetrate. Hence, nanocrystals, which typically possess a size >200 nm, will not enter the skin after dermal application [106,107,108,109,110,111]. Instead, they remain in the vehicle on top of the skin, and only dissolved molecules that are released from the nanocrystals can penetrate into the skin (c.f. Figure 1 and Figure 2). However, after dermal application, the vehicle, for example, a cream or a gel, will change its properties. Water will evaporate over time, and oil droplets might coalesce [112,113]. These changes in the vehicle will also change the distribution of the nanocrystals within the vehicle and/or their interaction with the skin. These effects are very complex and vary depending on the type and amount of vehicle used. As a result, predicting the fate of nanocrystals following dermal application, in general, is not possible and needs to be determined separately for each formulation.

## 3. Benefits of Nanocrystals for Dermal Application

Dermal application of active compounds can have different purposes, depending on the target site to which the ingredients need to be delivered. In pharmaceutics, transdermal penetration of the active ingredient is often desired. In cosmetics, the transport of the active compound should be either into the skin or onto its surface. Targeted dermal drug delivery is necessary to achieve this, and it can only be accomplished by understanding the mechanisms by which active ingredients are transported into the skin [114,115].

In principle, there are three major pathways through which active ingredients can enter the skin. The first mechanism is passive diffusion. This means that the active ingredient must be dissolved in the vehicle, from which it diffuses to the skin surface and then into the skin. This principle is explained by Fick’s first law [8,116] as in Equation (2).

(2) $$J=\frac{D\times (C_{1}-C_{2})}{h}$$

where

- *J* is the steady-state flux (the rate at which the drug permeates through the membrane per unit area per unit time, e.g., mg/cm^2^/h).
- *D* is the diffusion coefficient of the drug.
- *C*_1_ is the concentration of the drug on the donor side of the membrane (outermost part of the stratum corneum from which the drug is released).
- *C*_2_ is the concentration of the drug on the receptor side of the membrane (the inner layer of the stratum corneum where the drug is being absorbed).
- *h* is the thickness of the membrane or skin.

Based on this, it is evident that a higher amount of dissolved active ingredient can increase the dermal penetration of active compounds [117,118]. Consequently, nanocrystals—which increase the solubility—can be used to enhance the passive dermal penetration of poorly water-soluble active ingredients, as demonstrated by several studies [1,70,77,88].

**Figure 3:**
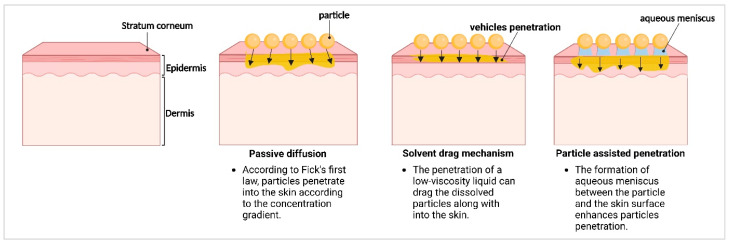
Schematic representation of active compound’s penetration mechanisms into the skin.

Another mechanism by which active compounds penetrate the skin that was recently found is particle-assisted dermal penetration [119]. This means that the addition of particles to a formulation in which active ingredients are dissolved leads to an increase in the dermal penetration efficacy (total amount of penetrated active ingredient and penetration depth) of the active ingredient being dissolved in the vehicle. The reason for this is that the particles attach to the skin, creating a meniscus between the skin and the particle (Figure 3). The meniscus is made up of the vehicle and contains the dissolved ingredient. The retention time of the particles is considered to be much longer than that of the vehicle. Therefore, the apparent penetration time of the active ingredient increases, which then increases the penetration efficacy. The adherence of the particles to the skin and the prolonged contact with the vehicle meniscus also locally enhance skin hydration, which further fosters the dermal penetration efficacy [119,120] (Figure 3).

The penetration-improving effect of particles is concentration- and size-dependent. In particular, particle-assisted dermal penetration is higher with greater amounts of particles and smaller particle sizes within the formulation [120]. Particle-assisted dermal penetration can be achieved by adding nanocrystals to formulations well above the saturation solubility of the active compound. This effect was already shown for nanocrystals in 2016 [78] and subsequently understood and confirmed in 2022 [119].

Another recently found mechanism is the solvent drag mechanism, whereby liquids applied to the skin can penetrate the skin, and the active ingredients dissolved in the liquid are dragged into the skin along with their solvent [121]. Nanocrystals enhance the solubility of poorly water-soluble drugs, allowing more active substance to dissolve in the solvent. If the solvent (e.g., water) penetrates the skin, the dermal penetration of the active ingredient can be enhanced. To utilize the solvent drag mechanism, formulations that provide high amounts of liquid that can penetrate the skin and in which the active ingredient is dissolved should be used. This can be achieved, for example, with the use of thixotropic gels that transform into liquids upon application on the skin [90].

Summarizing penetration into the skin, nanocrystals have been proven to be useful in enhancing the dermal penetration efficacy of poorly soluble active ingredients by using three different penetration mechanisms: passive diffusion, particle-assisted dermal penetration, and the solvent drag mechanism. The latter two mechanisms are most effective if higher concentrations of nanocrystals, exceeding the saturation solubility of the poorly soluble active compound, are added to the formulation. In this way, nanocrystals can also improve the penetration of compounds dissolved in the vehicle. In this case, nanocrystals would provide particle-assisted dermal penetration for all such molecules. Hence, improved co-delivery of active compounds can also be achieved with these nanocrystals. This approach is highly promising, particularly for the co-delivery of anti-inflammatory and analgesic drugs, as well as for the simultaneous delivery of different types of antibiotics, among other applications.

### 3.1. Nanocrystals for Hair Follicle Targeting

**Figure 4:**
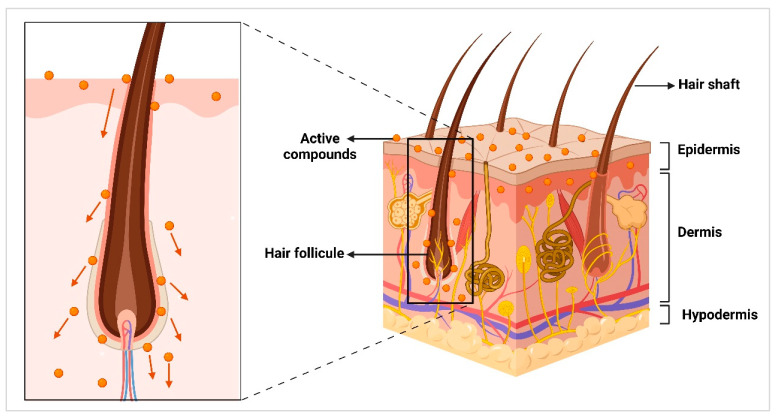
Penetration of active compounds into the viable dermis (left, arrows) via the hair follicle route.

Transporting active ingredients via hair follicles into or through the skin is another interesting route of dermal application for nanocrystals. The uptake of molecules into hair follicles requires particles that are preferably in the size range between 500 and 800 nm. This particle size range fits optimally into the tiny structures of the hair surface and can be transported into the hair via the ratchet mechanism while the hair is moving (Figure 4). This mechanism was proposed by Prof. Jürgen Lademann and his co-workers at Charité in Berlin, Germany, and has since been proven in many other studies [92,122,123,124,125,126].

The milling process of nanocrystals (c.f. Section 2.2) can be adopted, enabling the production of nanocrystals with tailor-made particle sizes. Therefore, it is possible to easily produce nanocrystals with sizes optimal for hair follicle targeting. The advantage of nanocrystals over other nanosized drug carriers is that they are composed of 100% active material, whereas other drug nanocarriers are composed of a matrix or scaffold in which the active material is encapsulated. Typical loading capacities for such nanocarriers are between 1 and 10%. Hence, drug targeting into hair follicles is much more efficient with nanocrystals (composed of 100% active ingredient) than with other carriers that contain only 1–10% active ingredients. The effectiveness of nanocrystals for targeting active ingredients into the hair follicles has been shown in various studies [70,90,127,128,129].

Interestingly, a recent study showed that nanocrystals not only penetrate into hair follicles but can also facilitate the transport of other molecules dissolved in the dispersion medium of the nanocrystals [130]. This finding now opens the possibility to transport different types of active compounds into the hair follicles at the same time. Poorly soluble compounds can be transported into the hair follicle as nanocrystals, and freely soluble compounds can be dissolved in the dispersion medium. Once the active compounds are in the hair follicle, they can easily penetrate into the viable dermis, because the stratum corneum in the hair follicle is thinner and tighter than the stratum corneum on the skin surface (Figure 4). While the penetration of the dissolved active molecules from hair follicles into the skin exhibits penetration, it can be delayed and retarded for several days from the nanocrystals [131,132,133,134,135,136,137].

### 3.2. Nanocrystals and Microneedles

**Figure 5:**
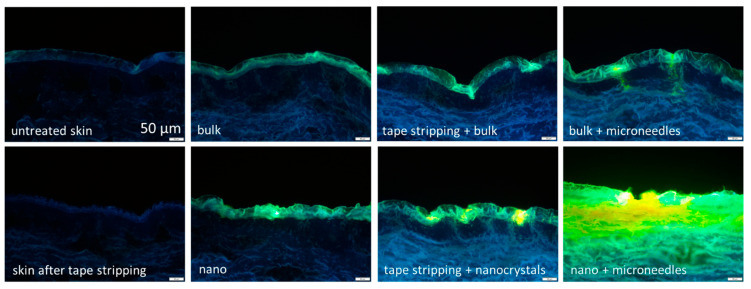
Visualizing the combined effect of nanocrystals and microneedle devices with images from vertical skin sections (porcine skin) obtained by inverted epifluorescence microscopy, in which green fluorescence indicates penetration of nanocrystals. Nanocrystals improve the dermal penetration of curcumin in comparison to larger-sized bulk material with a size of about 10 µm. Also, manipulation of the skin improves the dermal penetration. However, combining nanocrystal technology with microneedles resulted in a synergistic dermal delivery enhancement [138,139].

A further use for nanocrystals for enhanced dermal application is the co-administration of nanocrystals with microneedles. Microneedles create small holes in the skin barrier, allowing liquids to penetrate easily into deeper layers of the skin. However, poorly soluble compounds that are incorporated into the formulation as micrometer particles cannot penetrate through these holes. Hence, the co-administration of microneedles cannot improve the dermal penetration efficacy for such formulations. In contrast, nanocrystals are small enough to penetrate through the tiny channels created by the microneedles and can therefore be used for improved delivery of poorly soluble active compounds (Figure 5). Similarly to nanocrystals that enter the hair follicles, nanocrystals can enter the skin via the microchannels that are created by the microneedles, where they accumulate, forming a depot from which the active compound is slowly released while the nanocrystals dissolve [138,139].

### 3.3. Current State of Nanocrystals

Many pharmaceutical products that use nanocrystals for improved solubility and bioavailability of poorly water-soluble active compounds are already available on the market. Notably, most of these products are intended for oral administration [66,68]. However, only one pharmaceutical product using nanocrystal technology is available for dermal therapy. Nucryst^®^, a nanocrystal-based product by Nucryst Pharmaceuticals (Princeton, NJ, USA), utilizes antimicrobial silver nanocrystal technology and is intended to reduce skin inflammation and microbial activity [66,67,91]. Furthermore, a variety of cosmetic products utilizing nanocrystal technology are currently available on the market. In 2007, the first cosmetic products with rutin were launched in the markets; they were Juvedical products (Age-Decoder Face Fluid and Face Cream, Juvena, Switzerland). Another rutin-based nanocrystal product in the market is the product Edelweiss (Audorasan, Regensburg, Germany). Also, Platinum Rare, a hesperidin-based nanocrystal formulation that is marketed by La Prairie Group AG (Zürich, Switzerland), is available in the markets as an anti-aging and rejuvenation cosmetic formulation [70,77]. Despite these products, the availability of marketed dermal nanocrystal products is limited and might be attributed to the higher market value of such cosmetic products and/or a still limited awareness of this simple but highly effective formulation principle. However, the benefits of nanocrystals for dermal application have been proven in numerous scientific studies. For instance, the implementation of nanocrystals to improve the efficacy of poorly water-soluble natural antioxidants such as resveratrol [140,141,142], apigenin [143], lutein [144], rutin [67,91,145,146], hesperidin [147,148], quercetin [149,150], and curcumin [70,81,90,151,152]. Another example is the increased water solubility of broad-spectrum filters (e.g., trisbiphenyl triazine (TBPT) and methylene bis-benzotriazolyl tetramethylbutylphenol (MBBT)), which can increase the loading capacity and efficacy of sunscreens [153]. Moreover, several drugs used to treat pain and inflammation, such as flurbiprofen and diclofenac, have been produced as nanocrystals, and studies have revealed that these two drugs offer enhanced skin penetration and delivery when formulated as nanocrystals [70,154]. Further studies showed the preparation of apremilast nanocrystals to treat psoriasis. Results of this study showed a greater saturation solubility than micronized apremilast and exhibited an increase in the drug distribution in the skin without adverse effects [70]. Additionally, nanocrystal formulations of fusidic acid showed an enhanced drug distribution and increased bacterial exposure in the infected wound in comparison to the coarse cream formulation [155]. Although a wide variety of topical nanocrystals formulations have shown the beneficial effects of nanocrystals in different dermal therapeutic treatments, many pharmaceutical products are still in the development or clinical trial phases [69,70,91]. Nonetheless, in the future, we can expect nanocrystals to enter the market not only for cosmetic purposes but also for pharmaceutical applications, with great success.

## 4. Future Perspectives

Since the invention of nanocrystals in the early 1990s, nanocrystal technology has been proven to be highly beneficial for improved solubility and bioavailability of poorly soluble active compounds, with many products currently available for oral and dermal cosmeceutical applications. With all this scientific evidence, the future will hopefully encourage the pharmaceutical industry to exploit this simple formulation principle more often in topical drug products. Future trends should include the use of nanocrystals in hair growth products (i.e., hair follicle targeting) or the use of nanocrystals with microneedle devices, for example, for efficient local pain treatment.

Another future perspective is the use of nanocrystal technology, namely the milling of larger-sized bulk material to below 1 µm, not only for isolated chemical compounds but also for natural multi-component materials. Nanocrystals composed of multi-component materials were first developed in 2012 [156]. These nanocrystals were produced from plants and plant parts and were named “plantCrystals”. They have different properties from the classical nanocrystals, offering more benefits and applications. Additional details about this novel and highly promising formulation principle are provided below.

### 4.1. PlantCrystals—Nanocrystals Derived from Plant Material

Plants and parts of plants have been used in nutrition, cosmetics, and health applications for thousands of years, serving as a sustainable and natural source of various bioactive compounds, many of which exhibit therapeutic properties such as antioxidant, anti-inflammatory, antimicrobial, wound healing, and analgesic activities [157,158,159,160,161]. In addition to the use of the whole-plant material, plant extraction is often employed to extract bioactive compounds from plants or plant materials to enhance their activity and bio-efficacy. Plant extraction is typically performed by traditional extraction methods such as maceration, percolation, decoction, and other techniques. These approaches often require the use of organic solvents and can be very time-consuming. Also, they typically require high amounts of plant starting material while yielding only small quantities of the bioactive compounds of interest and produce lots of organic waste material [162,163]. Therefore, modern extraction techniques that overcome these disadvantages are of high interest, and plantCrystals are among these strategies [164].

The first plant-derived nanocrystal formulation was a curcumin nanosuspension [156]. Curcumin has many beneficial health properties, but its bioavailability is poor due to its low solubility in water [165,166]. By that time, many studies had already shown the benefits of nanocrystals for improved bioactivity of curcumin [78,111,120,156,167]. However, the use of whole plant material was considered to offer more advantages [168,169]. The first comprehensive studies for milling plant materials were performed by Griffin et al., who showed that the milled plant material has a higher bio-efficacy than the bulk material and classical extracts. They also demonstrated that in this way it is possible to reuse plant waste by turning it into effective formulations and products [79,170,171].

**Figure 6:**
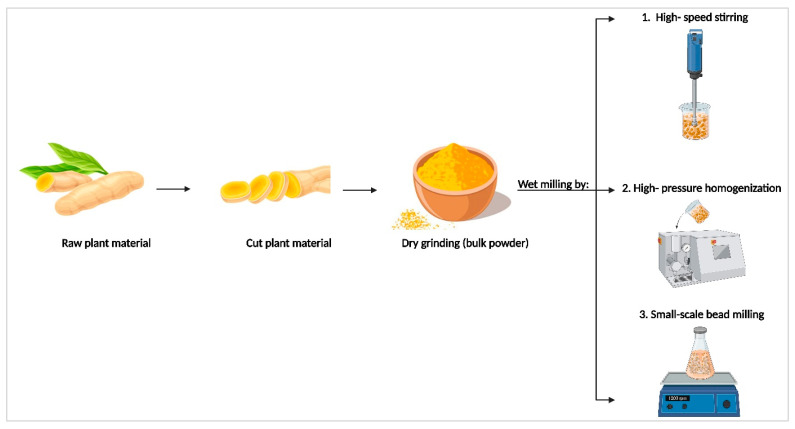
Scheme of process to produce plantCrystals by wet milling techniques.

The principle behind plantCrystals is not only the reduction in particle size. It is rather the destruction of the plant cells, which then release their content more efficiently. A typical plant cell has a size of about 10 µm. Consequently, milling the plant material to below 1 µm leads to destruction of all plant cells and most of the plant cell parts, such as cell walls and vacuoles. This allows a fast and exhaustive release of the active molecules from the disrupted plant cell. Classical plant extraction is the combination of two steps. Step one is the wash-out phase, which is rapid, and step two is the diffusion phase, where the actives diffuse out of the plant cells. The second step is time-consuming and is the reason why many classical plant extraction methods are not exhaustive, as not all active molecules can diffuse out of the cells. In contrast, in plantCrystal formulations, all plant cells are destroyed, allowing the active compounds to be washed out exhaustively, without the need for the second step. Consequently, plantCrystal technology can be regarded as a fast and exhaustive extraction method. Typically, the milling is performed in water. This means that, in comparison to classical methods where large quantities of organic solvents are used, plantCrystal technology represents a very sustainable and environmentally friendly technique. Similarly to nanocrystals, plantCrystals can be produced by using various wet milling techniques, such as bead milling, high-speed stirring, high-pressure homogenization, or a combination of these methods [93,95,98,168,172] (Figure 6).

**Figure 7:**
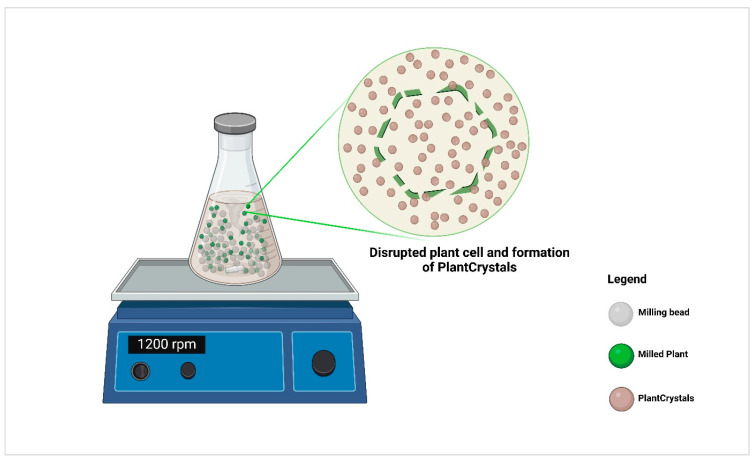
The principle of plantCrystal technology through a small-scale bead milling approach, representing the plant cell rupture and production of plantCrystals.

PlantCrystals are produced by dispersing the plant material, either in powder form or as an aqueous dispersion, into a stabilizer solution (e.g., surfactant) to prevent agglomeration over time [168]. The production can be performed on a laboratory scale or a large scale by using the same equipment used for the production of nanocrystals. For early-stage development, ultra-small batches can also be produced by using the small-scale bead milling technique. In this process, the dispersed plant material in the aqueous surfactant solution is combined with zirconium beads at a specific ratio and stirred using a magnetic stirrer. The mixture is rotated in a flask or vial at a fixed speed for a defined period of time [168,173] (Figure 7).

### 4.2. PlantCrystals for Improved Dermal Penetration

PlantCrystals have been shown to allow for an improved dermal penetration of their active ingredients [168,169,172]. The mechanisms of penetration enhancement are similar to those described for the nanocrystals (c.f. Section 3). In addition, one more mechanism was discovered in 2022, when it was found that the nano-milling process not only destroys the plant cells but also forms extracellular vesicles, i.e., exosomes. Plant-derived extracellular vesicles and exosomes are novel formulation principles that can be used to improve the bioavailability of active compounds [174,175,176,177]. However, the production of these vesicles is very tedious and time-consuming, requiring many purification steps and resulting in relatively low yields. In contrast, plantCrystals can be produced swiftly in a one-step process, require no purification, and as a result, the yield obtained is 100% [168,173]. So far, only a few studies have systematically investigated the benefits of plantCrystals for dermal application and compared their efficacy in enhancing dermal penetration to traditional formulation principles and extracellular vesicles. A very recent publication by Alkhaldi et al. demonstrated the superiority of plantCrystals, which increased the dermal penetration efficacy of the poorly water-soluble curcumin by approximately 2.7-fold compared to classical extracellular vesicles [178]. The superiority of plantCrystals over classical formulations and extracellular vesicles was also demonstrated by Sehra et al. [179]. In fact, plantCrystals can be regarded as a new generation of nanocrystals. They represent a novel, high-potential drug delivery system for improved dermal delivery of poorly water-soluble active compounds, as well as a sustainable and highly economical plant extraction method. Therefore, the use of plantCrystals in future dermal applications (pharmaceutical and cosmetic) is highly recommended.

## 5. Synopsis

In our opinion, nanocrystals are the formulation principle of choice to overcome issues associated with poor solubility. The potential of nanocrystal technology to improve the bioavailability of poorly soluble active compounds has been proven in many pharmaceutical products, mainly for oral application. However, the use of nanocrystals in pharmaceutical products for dermal applications has not yet been fully exploited. The reason might be the increased risk of physical instability during storage, as the nanocrystals need to be formulated in a liquid or semi-liquid state, which increases the risk of crystal growth due to Ostwald ripening or particle agglomeration. This risk can be reduced by producing nanocrystals with a narrow particle size and by adding protective colloids. It would also be possible to transfer the nanocrystals into a solid state, for example, into a freeze-dried powder that needs to be redispersed shortly prior to application.

Additionally, the possibility of formulating and producing effective and physically stable dermal nanocrystal products has been shown in many cosmetic products; therefore, it will also be possible to formulate and produce pharmaceutical nanocrystal products for topical application with sufficient pharmaceutical quality.

We suggest that the most promising strategies for nanocrystals in dermal products include using nanocrystals to enhance the passive diffusion of poorly soluble actives. The combination with microneedle treatment is recommended to achieve penetration of the nanocrystals into the viable dermis, where they can form a depot for long-lasting effects. Moreover, it is highly advisable to exploit nanocrystals for the delivery of active compounds to the hair follicles. Effective and safe treatment of hair loss would be one of the unmet needs that could be addressed very efficiently with nanocrystal technology, particularly by using nanocrystals with sizes around 600 nm.

PlantCrystals are plant-based nanocrystals and represent a novel, promising, and sustainable strategy for improved extraction and bio-efficacy of plant materials. During the production of plantCrystals, exosomes are formed, which can be used to enhance the dermal penetration of active compounds from the plant from which they are derived. They can also be used as carriers to improve the bioactivity of other active compounds, which can be added to the plantCrystals during their production. PlantCrystal extracts contain no organic solvents and are thus not harmful to the skin. They can be directly incorporated into dermal products to formulate highly efficient, natural, and skin-friendly solutions.

Due to the increased consumer demand for green technology-based formulations that offer natural and biocompatible skincare products, it is expected that the next decade will likely see significant advancements in plantCrystal technology as a formulation strategy, leading to the successful translation of environmentally friendly products targeted for dermal therapeutics. However, plantCrystals are still in the investigation phase, with only a few research studies available. Thus, there are currently no commercially available plantCrystal products. For this reason, comprehensive research in this field is suggested.

Finally, for a successful transfer of nanocrystal technology into pharmaceutical products, close collaboration between academia, dermatological experts, and pharmaceutical industries is essential to overcome the gap between research and market translation, enabling laboratory findings to be translated into optimized, large-scale, commercially available products.

## 6. Conclusions

Drug nanocrystals are a powerful formulation strategy to overcome the issues associated with poorly soluble active compounds. The technology was invented in the early 1990s, and many pharmaceutical products are already available for oral and intravenous applications. However, dermal pharmaceutical products are not yet available, although the benefits of nanocrystals for dermal application have been demonstrated in numerous scientific studies and in various marketed cosmetic products that are already on the market. Based on the success of cosmetic nanocrystals and the insights gained from them, the path for the development of pharmaceutical nanocrystals has been paved.

## Data Availability

No new data were created or analyzed in this study. Data sharing is not applicable to this article.

## References

[1] Parmar P.K., Wadhawan J., Bansal A.K. (2021). Pharmaceutical nanocrystals: A promising approach for improved topical drug delivery. Drug Discov. Today.

[2] Tapfumaneyi P., Imran M., Mohammed Y., Roberts M.S. (2022). Recent advances and future prospective of topical and transdermal delivery systems. Front. Drug Deliv..

[3] Jaiswal D., Jain P. (2023). Recent Updates and Advancement of Transdermal Drug Delivery System. IJSRSET.

[4] Jorge L.L., Feres C.C., Teles V.E. (2010). Topical preparations for pain relief: Efficacy and patient adherence. J. Pain Res..

[5] Salatin S., Lotfipour F., Jelvehgari M. (2019). A brief overview on nano-sized materials used in the topical treatment of skin and soft tissue bacterial infections. Expert Opin. Drug Deliv..

[6] Eastman W.J., Malahias S., Delconte J., DiBenedetti D. (2014). Assessing Attributes of Topical Vehicles for the Treatment of Acne, Atopic Dermatitis, and Plaque Psoriasis. Cutis.

[7] Valarmathi S., Admuthe J.A., Pimpalshende P.M., Philip R., Solunke R.S., Bonde N.R., Meenakshi, Paria A. (2024). Formulation and Optimization of Topical Creams for Dermatological Disorders. Educ. Adm. Theory Pract..

[8] Cornier J., Keck C., van de Voorde M. (2019). Nanocosmetics: From Ideas to Products.

[9] Draelos Z.D. (2021). Cosmetic Dermatology: Products and Procedures.

[10] Gupta V., Mohapatra S., Mishra H., Farooq U., Kumar K., Ansari M.J., Aldawsari M.F., Alalaiwe A.S., Mirza M.A., Iqbal Z. (2022). Nanotechnology in Cosmetics and Cosmeceuticals-A Review of Latest Advancements. Gels.

[11] Elbouzidi A., Haddou M., Baraich A., Taibi M., El Hachlafi N., Pareek A., Mesnard F., Addi M. (2025). Biochemical insights into specialized plant metabolites: Advancing cosmeceutical applications for skin benefits. J. Agric. Food Res..

[12] Gao X.-H., Zhang L., Wei H., Chen H.-D. (2008). Efficacy and safety of innovative cosmeceuticals. Clin. Dermatol..

[13] Resende D.I.S.P., Ferreira M.S., Lobo J.M.S., Sousa E., Almeida I.F. (2022). Skin Depigmenting Agents in Anti-Aging Cosmetics: A Medicinal Perspective on Emerging Ingredients. Appl. Sci..

[14] Xie M., Jiang Z., Lin X., Wei X. (2024). Application of plant extracts cosmetics in the field of anti-aging. J. Dermatol. Sci. Cosmet. Technol..

[15] Sabalingam S., Siriwardhene M.A. (2022). A review on emerging applications of emulgel as topical drug delivery system. World J. Adv. Res. Rev..

[16] Talat M., Zaman M., Khan R., Jamshaid M., Akhtar M., Mirza A.Z. (2021). Emulgel: An effective drug delivery system. Drug Dev. Ind. Pharm..

[17] Gómez-Farto A., Jiménez-Escobar A.L., Pérez-González N., Castán H., Clares B., Arias-Santiago S., Montero-Vílchez T. (2024). Development of an Emulgel for the Effective Treatment of Atopic Dermatitis: Biocompatibility and Clinical Investigation. Gels.

[18] Bodmer T., Hartmann S.F., Keck C.M., Kleiner M., Köhler K. (2023). Production of Hydrogel-Based Curcumin-Loaded O/W Suspoemulsions. Future Pharmacol..

[19] Kumar D., Sil D., Kurmi B.D., Kumar M. (2025). Future Prospects and Regulatory Pathways for Invasome Technologies in Transdermal Drug Delivery. Assay Drug Dev. Technol..

[20] Fan L., Huang J., Ma S. (2024). Recent advances in delivery of transdermal nutrients: A review. Exp. Dermatol..

[21] Sim Y.S., Wong L.C., Yeoh S.C., Almashhadani A., Alrimawi B.H., Goh C.F. (2025). Skin penetration enhancers: Mechanistic understanding and their selection for formulation and design. Drug Deliv. Transl. Res..

[22] Lane M.E. (2013). Skin penetration enhancers. Int. J. Pharm..

[23] Panda P., Mohanty T., Mohapatra R. (2025). Advancements in Transdermal Drug Delivery Systems: Harnessing the Potential of Macromolecular Assisted Permeation Enhancement and Novel Techniques. AAPS Pharmscitech.

[24] Haque T., Talukder M.M.U. (2018). Chemical Enhancer: A Simplistic Way to Modulate Barrier Function of the Stratum Corneum. Adv. Pharm. Bull..

[25] Kirjavainen M., Mönkkönen J., Saukkosaari M., Valjakka-Koskela R., Kiesvaara J., Urtti A. (1999). Phospholipids affect stratum corneum lipid bilayer fluidity and drug partitioning into the bilayers. J. Control. Release.

[26] Ostróżka-Cieślik A. (2022). The Potential of Pharmaceutical Hydrogels in the Formulation of Topical Administration Hormone Drugs. Polymers.

[27] Almoshari Y. (2022). Novel Hydrogels for Topical Applications: An Updated Comprehensive Review Based on Source. Gels.

[28] Stan D., Tanase C., Avram M., Apetrei R., Mincu N.-B., Mateescu A.L., Stan D. (2021). Wound healing applications of creams and “smart” hydrogels. Exp. Dermatol..

[29] Abdullah H.M., Farooq M., Adnan S., Masood Z., Saeed M.A., Aslam N., Ishaq W. (2023). Development and evaluation of reservoir transdermal polymeric patches for controlled delivery of diclofenac sodium. Polym. Bull..

[30] Cheng A., Zhang S., Meng F., Xing M., Liu H., Yang G., Gao Y. (2024). Nanosuspension-Loaded Dissolving Microneedle Patches for Enhanced Transdermal Delivery of a Highly Lipophilic Cannabidiol. Int. J. Nanomed..

[31] Dumitriu Buzia O., Păduraru A.M., Stefan C.S., Dinu M., Cocoș D.I., Nwabudike L.C., Tatu A.L. (2023). Strategies for Improving Transdermal Administration: New Approaches to Controlled Drug Release. Pharmaceutics.

[32] Campani V., Scotti L., Silvestri T., Biondi M., de Rosa G. (2020). Skin permeation and thermodynamic features of curcumin-loaded liposomes. J. Mater. Sci. Mater. Med..

[33] Nsairat H., Khater D., Sayed U., Odeh F., Al Bawab A., Alshaer W. (2022). Liposomes: Structure, composition, types, and clinical applications. Heliyon.

[34] Schlich M., Musazzi U.M., Campani V., Biondi M., Franzé S., Lai F., de Rosa G., Sinico C., Cilurzo F. (2022). Design and development of topical liposomal formulations in a regulatory perspective. Drug Deliv. Transl. Res..

[35] Kim M.-H., Jeon Y.-E., Kang S., Lee J.-Y., Lee K.W., Kim K.-T., Kim D.-D. (2020). Lipid Nanoparticles for Enhancing the Physicochemical Stability and Topical Skin Delivery of Orobol. Pharmaceutics.

[36] Chantaburanan T., Teeranachaideekul V., Jintapattanakit A., Chantasart D., Junyaprasert V.B. (2023). Enhanced stability and skin permeation of ibuprofen-loaded solid lipid nanoparticles based binary solid lipid matrix: Effect of surfactant and lipid compositions. Int. J. Pharm. X.

[37] Almawash S. (2023). Solid lipid nanoparticles, an effective carrier for classical antifungal drugs. Saudi Pharm. J..

[38] Chutoprapat R., Kopongpanich P., Chan L.W. (2022). A Mini-Review on Solid Lipid Nanoparticles and Nanostructured Lipid Carriers: Topical Delivery of Phytochemicals for the Treatment of Acne Vulgaris. Molecules.

[39] Argenziano M., Haimhoffer A., Bastiancich C., Jicsinszky L., Caldera F., Trotta F., Scutera S., Alotto D., Fumagalli M., Musso T. (2019). In Vitro Enhanced Skin Permeation and Retention of Imiquimod Loaded in β-Cyclodextrin Nanosponge Hydrogel. Pharmaceutics.

[40] Riccio B.V.F., Meneguin A.B., Baveloni F.G., de Antoni J.A., Robusti L.M.G., Gremião M.P.D., Ferrari P.C., Chorilli M. (2023). Biopharmaceutical and nanotoxicological aspects of cyclodextrins for non-invasive topical treatments: A critical review. J. Appl. Toxicol..

[41] Soe H.M.S.H., Maw P.D., Loftsson T., Jansook P. (2022). A Current Overview of Cyclodextrin-Based Nanocarriers for Enhanced Antifungal Delivery. Pharmaceuticals.

[42] Topuz F., Uyar T. (2024). Recent Advances in Cyclodextrin-Based Nanoscale Drug Delivery Systems. Wiley Interdiscip. Rev. Nanomed. Nanobiotechnol..

[43] Souto E.B., Cano A., Martins-Gomes C., Coutinho T.E., Zielińska A., Silva A.M. (2022). Microemulsions and Nanoemulsions in Skin Drug Delivery. Bioengineering.

[44] Roy A., Nishchaya K., Rai V.K. (2022). Nanoemulsion-based dosage forms for the transdermal drug delivery applications: A review of recent advances. Expert Opin. Drug Deliv..

[45] Preeti, Sambhakar S., Malik R., Bhatia S., Al Harrasi A., Rani C., Saharan R., Kumar S., Geeta, Sehrawat R. (2023). Nanoemulsion: An Emerging Novel Technology for Improving the Bioavailability of Drugs. Scientifica.

[46] Chatterjee S., Hui P.C.-L. (2021). Review of Applications and Future Prospects of Stimuli-Responsive Hydrogel Based on Thermo-Responsive Biopolymers in Drug Delivery Systems. Polymers.

[47] van Gheluwe L., Chourpa I., Gaigne C., Munnier E. (2021). Polymer-Based Smart Drug Delivery Systems for Skin Application and Demonstration of Stimuli-Responsiveness. Polymers.

[48] Bellarmin M., Nandhini J., Karthikeyan E., Mahalakshmi D., Karthik K.K. (2025). A Comprehensive Review on Stimuli-Responsive Nanomaterials: Advancements in Wound Healing and Tissue Regeneration. Biomed. Mater. Devices.

[49] Iachina I., Eriksson A.H., Bertelsen M., Petersson K., Jansson J., Kemp P., Engell K.M., Brewer J.R., Nielsen K.T. (2023). Dissolvable microneedles for transdermal drug delivery showing skin pentation and modified drug release. Eur. J. Pharm. Sci..

[50] Priya S., Tomar Y., Desai V.M., Singhvi G. (2023). Enhanced skin drug delivery using dissolving microneedles: A potential approach for the management of skin disorders. Expert Opin. Drug Deliv..

[51] Kheirieh A., Abbasi A., Malaekeh-Nikouei B., Golmohammadzadeh S., Mousavi Shaegh S.A. (2025). Delivery of nanocarriers with dissolvable microneedles for skin treatments: Approaches and challenges. J. Drug Deliv. Sci. Technol..

[52] de Oliveira R.S., Fantaus S.S., Guillot A.J., Melero A., Beck R.C.R. (2021). 3D-Printed Products for Topical Skin Applications: From Personalized Dressings to Drug Delivery. Pharmaceutics.

[53] Tsegay F., Elsherif M., Butt H. (2022). Smart 3D Printed Hydrogel Skin Wound Bandages: A Review. Polymers.

[54] Uchida D.T., Bruschi M.L. (2023). 3D Printing as a Technological Strategy for the Personalized Treatment of Wound Healing. AAPS Pharmscitech.

[55] Brown M.B., Martin G.P., Jones S.A., Akomeah F.K. (2006). Dermal and transdermal drug delivery systems: Current and future prospects. Drug Deliv..

[56] Hasanpour F., Budai-Szűcs M., Kovács A., Ambrus R., Jójárt-Laczkovich O., Cseh M., Geretovszky Z., Ayaydin F., Berkó S. (2024). Improvement of lidocaine skin permeation by using passive and active enhancer methods. Int. J. Pharm..

[57] Münch S., Wohlrab J., Neubert R.H.H. (2017). Dermal and transdermal delivery of pharmaceutically relevant macromolecules. Eur. J. Pharm. Biopharm..

[58] Jhanker Y., Mbano M.N., Ponto T., Espartero L.J.L., Yamada M., Prow T., Benson H.A.E. (2021). Comparison of physical enhancement technologies in the skin permeation of methyl amino levulinic acid (mALA). Int. J. Pharm..

[59] Trommer H., Neubert R.H.H. (2006). Overcoming the stratum corneum: The modulation of skin penetration. A review. Skin Pharmacol. Physiol..

[60] Wang Y., Zeng L., Song W., Liu J. (2022). Influencing factors and drug application of iontophoresis in transdermal drug delivery: An overview of recent progress. Drug Deliv. Transl. Res..

[61] Wu X.-M., Todo H., Sugibayashi K. (2007). Enhancement of skin permeation of high molecular compounds by a combination of microneedle pretreatment and iontophoresis. J. Control. Release.

[62] Wang J.V., Friedman P.M., Rodeberg D., Konda A., Parker C., Geronemus R.G. (2022). Enhancing Skin Uptake of Topical Antioxidants With 1,440-nm Nonablative Fractional Diode Laser Pretreatment. Dermatol. Surg..

[63] Wang J.V., Friedman P.M., Agron S., Konda A., Parker C., Geronemus R.G. (2022). Quantifying Skin Uptake of Topicals After 1,927-nm and 1,440-nm Nonablative Fractional Diode Laser Treatment. Dermatol. Surg..

[64] Escobar-Chávez J.J., Bonilla-Martínez D., Villegas-González M.A., Revilla-Vázquez A.L. (2009). Electroporation as an efficient physical enhancer for skin drug delivery. J. Clin. Pharmacol..

[65] Scott J.A., Banga A.K. (2015). Cosmetic devices based on active transdermal technologies. Ther. Deliv..

[66] Junghanns J.-U.A.H., Müller R.H. (2008). Nanocrystal technology, drug delivery and clinical applications. Int. J. Nanomed..

[67] Shegokar R., Müller R.H. (2010). Nanocrystals: Industrially feasible multifunctional formulation technology for poorly soluble actives. Int. J. Pharm..

[68] Chary P.S., Shaikh S., Bhavana V., Rajana N., Vasave R., Mehra N.K. (2024). Emerging role of nanocrystals in pharmaceutical applications: A review of regulatory aspects and drug development process. Appl. Mater. Today.

[69] Liu Y., Zhao J., Chen J., Miao X. (2023). Nanocrystals in cosmetics and cosmeceuticals by topical delivery. Colloids Surf. B Biointerfaces.

[70] Alnaim A.S. (2024). Nanocrystals in Dermal Drug Delivery: A Breakthrough for Enhanced Skin Penetration and Targeted Skin Disorder Treatments. Pharmaceutics.

[71] Jarvis M., Krishnan V., Mitragotri S. (2019). Nanocrystals: A perspective on translational research and clinical studies. Bioeng. Transl. Med..

[72] Chogale M.M., Ghodake V.N., Patravale V.B. (2016). Performance Parameters and Characterizations of Nanocrystals: A Brief Review. Pharmaceutics.

[73] Gigliobianco M.R., Casadidio C., Censi R., Di Martino P. (2018). Nanocrystals of Poorly Soluble Drugs: Drug Bioavailability and Physicochemical Stability. Pharmaceutics.

[74] Lhaglham P., Jiramonai L., Jia Y., Huang B., Huang Y., Gao X., Zhang J., Liang X.-J., Zhu M. (2024). Drug nanocrystals: Surface engineering and its applications in targeted delivery. iScience.

[75] Sun L., Xiang H., Ge C., Chen X., Zhang Q., Zhang Y., Miao X. (2021). A Nanocrystals-Based Topical Drug Delivery System with Improved Dermal Penetration and Enhanced Treatment of Skin Diseases. J. Biomed. Nanotechnol..

[76] Singh V., Bansal K., Bhati H., Bajpai M. (2024). New Insights into Pharmaceutical Nanocrystals for the Improved Topical Delivery of Therapeutics in Various Skin Disorders. Curr. Pharm. Biotechnol..

[77] Pelikh O., Stahr P.-L., Huang J., Gerst M., Scholz P., Dietrich H., Geisel N., Keck C.M. (2018). Nanocrystals for improved dermal drug delivery. Eur. J. Pharm. Biopharm..

[78] Vidlářová L., Romero G.B., Hanuš J., Štěpánek F., Müller R.H. (2016). Nanocrystals for dermal penetration enhancement—Effect of concentration and underlying mechanisms using curcumin as model. Eur. J. Pharm. Biopharm..

[79] Griffin S., Masood M.I., Nasim M.J., Sarfraz M., Ebokaiwe A.P., Schäfer K.-H., Keck C.M., Jacob C. (2017). Natural Nanoparticles: A Particular Matter Inspired by Nature. Antioxidants.

[80] Khan B.A., Rashid F., Khan M.K., Alqahtani S.S., Sultan M.H., Almoshari Y. (2021). Fabrication of Capsaicin Loaded Nanocrystals: Physical Characterizations and In Vivo Evaluation. Pharmaceutics.

[81] Ruggeri M., Sánchez-Espejo R., Casula L., Barbosa R.d.M., Sandri G., Cardia M.C., Lai F., Viseras C. (2022). Clay-Based Hydrogels as Drug Delivery Vehicles of Curcumin Nanocrystals for Topical Application. Pharmaceutics.

[82] Wang Y., Tan X., Fan X., Zhao L., Wang S., He H., Yin T., Zhang Y., Tang X., Jian L. (2021). Current strategies for oral delivery of BCS IV drug nanocrystals: Challenges, solutions and future trends. Expert Opin. Drug Deliv..

[83] Chen M.-L., John M., Lee S.L., Tyner K.M. (2017). Development Considerations for Nanocrystal Drug Products. AAPS J..

[84] Ye X., Patil H., Feng X., Tiwari R.V., Lu J., Gryczke A., Kolter K., Langley N., Majumdar S., Neupane D. (2016). Conjugation of Hot-Melt Extrusion with High-Pressure Homogenization: A Novel Method of Continuously Preparing Nanocrystal Solid Dispersions. AAPS PharmSciTech.

[85] Lai F., Pireddu R., Corrias F., Fadda A.M., Valenti D., Pini E., Sinico C. (2013). Nanosuspension improves tretinoin photostability and delivery to the skin. Int. J. Pharm..

[86] Shi Y., Wan A., Shi Y., Zhang Y., Chen Y. (2014). Experimental and mathematical studies on the drug release properties of aspirin loaded chitosan nanoparticles. Biomed Res. Int..

[87] Skinner L.M., Samble J.R. (1972). The Kelvin equation—A review. J. Aerosol Sci..

[88] Parveen N., Abourehab M.A.S., Thanikachalam P.V., Khar R.K., Kesharwani P. (2023). Nanocrystals as an emerging nanocarrier for the management of dermatological diseases. Colloids Surf. B Biointerfaces.

[89] Müller R.H., Gohla S., Keck C.M. (2011). State of the art of nanocrystals—Special features, production, nanotoxicology aspects and intracellular delivery. Eur. J. Pharm. Biopharm..

[90] Pelikh O., Eckert R.W., Pinnapireddy S.R., Keck C.M. (2021). Hair follicle targeting with curcumin nanocrystals: Influence of the formulation properties on the penetration efficacy. J. Control. Release.

[91] Patel V., Sharma O.P., Mehta T. (2018). Nanocrystal: A novel approach to overcome skin barriers for improved topical drug delivery. Expert Opin. Drug Deliv..

[92] Breuckmann P., Meinke M.C., Jaenicke T., Krutmann J., Rasulev U., Keck C.M., Müller R.H., Klein A.L., Lademann J., Patzelt A. (2021). Influence of nanocrystal size on the in vivo absorption kinetics of caffeine after topical application. Eur. J. Pharm. Biopharm..

[93] Keck C.M., Müller R.H. (2006). Drug nanocrystals of poorly soluble drugs produced by high pressure homogenisation. Eur. J. Pharm. Biopharm..

[94] Peltonen L., Hirvonen J. (2010). Pharmaceutical nanocrystals by nanomilling: Critical process parameters, particle fracturing and stabilization methods. J. Pharm. Pharmacol..

[95] Romero G.B., Keck C.M., Müller R.H. (2016). Simple low-cost miniaturization approach for pharmaceutical nanocrystals production. Int. J. Pharm..

[96] Chang T.-L., Zhan H., Liang D., Liang J.F. (2015). Nanocrystal technology for drug formulation and delivery. Front. Chem. Sci. Eng..

[97] Mohammad I.S., Hu H., Yin L., He W. (2019). Drug nanocrystals: Fabrication methods and promising therapeutic applications. Int. J. Pharm..

[98] Nakach M., Authelin J.-R., Perrin M.-A., Lakkireddy H.R. (2018). Comparison of high pressure homogenization and stirred bead milling for the production of nano-crystalline suspensions. Int. J. Pharm..

[99] Nakach M., Authelin J.-R., Agut C. (2017). New Approach and Practical Modelling of Bead Milling Process for the Manufacturing of Nanocrystalline Suspensions. J. Pharm. Sci..

[100] Yadav K.S., Kale K. (2020). High Pressure Homogenizer in Pharmaceuticals: Understanding Its Critical Processing Parameters and Applications. J. Pharm. Innov..

[101] Li J., Wang Z., Zhang H., Gao J., Zheng A. (2021). Progress in the development of stabilization strategies for nanocrystal preparations. Drug Deliv..

[102] Malamatari M., Taylor K.M.G., Malamataris S., Douroumis D., Kachrimanis K. (2018). Pharmaceutical nanocrystals: Production by wet milling and applications. Drug Discov. Today.

[103] Salazar J., Müller R.H., Möschwitzer J.P. (2014). Combinative Particle Size Reduction Technologies for the Production of Drug Nanocrystals. J. Pharm..

[104] Zhai X., Lademann J., Keck C.M., Müller R.H. (2014). Dermal nanocrystals from medium soluble actives—Physical stability and stability affecting parameters. Eur. J. Pharm. Biopharm..

[105] Tomić I., Juretić M., Jug M., Pepić I., Čižmek B.C., Filipović-Grčić J. (2019). Preparation of in situ hydrogels loaded with azelaic acid nanocrystals and their dermal application performance study. Int. J. Pharm..

[106] Prow T.W., Grice J.E., Lin L.L., Faye R., Butler M., Becker W., Wurm E.M.T., Yoong C., Robertson T.A., Soyer H.P. (2011). Nanoparticles and microparticles for skin drug delivery. Adv. Drug Deliv. Rev..

[107] Filon F.L., Mauro M., Adami G., Bovenzi M., Crosera M. (2015). Nanoparticles skin absorption: New aspects for a safety profile evaluation. Regul. Toxicol. Pharmacol..

[108] Nakazato G., Lonni A.A.S.G., Panagio L.A., de Camargo L.C., Gonçalves M.C., Reis G.F., Miranda-Sapla M.M., Tomiotto-Pellissier F., Kobayashi R.K.T., Rai M. (2020). Applications of Nanometals in Cutaneous Infections. Nanotechnology in Skin, Soft Tissue, and Bone Infections.

[109] Xiang H., Xu S., Li J., Pan S., Miao X. (2022). Particle Size Effect of Curcumin Nanocrystals on Transdermal and Transfollicular Penetration by Hyaluronic Acid-Dissolving Microneedle Delivery. Pharmaceuticals.

[110] Yao S., Chen N., Sun X., Wang Q., Li M., Chen Y. (2023). Size-dependence of the skin penetration of andrographolide nanosuspensions: In vitro release-ex vivo permeation correlation and visualization of the delivery pathway. Int. J. Pharm..

[111] Xiang H., Xu S., Zhang W., Li Y., Zhou Y., Miao X. (2023). Skin permeation of curcumin nanocrystals: Effect of particle size, delivery vehicles, and permeation enhancer. Colloids Surf. B Biointerfaces.

[112] Aranberri I., Binks B.P., Clint J.H., Fletcher P.D.I. (2004). Evaporation Rates of Water from Concentrated Oil-in-Water Emulsions. Langmuir.

[113] Santos O., Camargo M.F., Boock K., Bergamaschi M., Rocha Filho P. (2009). Analysis of the Phase Changes During Evaporation of Emulsions with Different Oil Phases. J. Dispers. Sci. Technol..

[114] Supe S., Takudage P. (2021). Methods for evaluating penetration of drug into the skin: A review. Skin Res. Technol..

[115] Gaikwad S.S., Zanje A.L., Somwanshi J.D. (2024). Advancements in transdermal drug delivery: A comprehensive review of physical penetration enhancement techniques. Int. J. Pharm..

[116] Hansen S., Lehr C.-M., Schaefer U.F. (2013). Improved input parameters for diffusion models of skin absorption. Adv. Drug Deliv. Rev..

[117] Liu C., Quan P., Fang L. (2016). Effect of drug physicochemical properties on drug release and their relationship with drug skin permeation behaviors in hydroxyl pressure sensitive adhesive. Eur. J. Pharm. Sci..

[118] Tapfumaneyi P., Imran M., Alavi S.E., Mohammed Y. (2023). Science of, and insights into, thermodynamic principles for dermal formulations. Drug Discov. Today.

[119] Wiemann S., Keck C.M. (2022). Particle-Assisted Dermal Penetration—A Simple Formulation Strategy to Foster the Dermal Penetration Efficacy. Pharmaceutics.

[120] Chaiprateep E.-O., Wiemann S., Eckert R.W., Raab C., Sengupta S., Keck C.M. (2023). Influence of Dose, Particle Size and Concentration on Dermal Penetration Efficacy of Curcumin. Pharmaceutics.

[121] Kaushik V., Ganashalingam Y., Schesny R., Raab C., Sengupta S., Keck C.M. (2021). Influence of Massage and Skin Hydration on Dermal Penetration Efficacy of Nile Red from Petroleum Jelly-An Unexpected Outcome. Pharmaceutics.

[122] Patzelt A., Mak W.C., Jung S., Knorr F., Meinke M.C., Richter H., Rühl E., Cheung K.Y., Tran N.B.N.N., Lademann J. (2017). Do nanoparticles have a future in dermal drug delivery?. J. Control. Release.

[123] Limcharoen B., Toprangkobsin P., Banlunara W., Wanichwecharungruang S., Richter H., Lademann J., Patzelt A. (2019). Increasing the percutaneous absorption and follicular penetration of retinal by topical application of proretinal nanoparticles. Eur. J. Pharm. Biopharm..

[124] Busch L., Keziban Y., Dähne L., Keck C.M., Meinke M.C., Lademann J., Patzelt A. (2021). The impact of skin massage frequency on the intrafollicular transport of silica nanoparticles: Validation of the ratchet effect on an ex vivo porcine skin model. Eur. J. Pharm. Biopharm..

[125] Lademann J., Patzelt A., Richter H., Antoniou C., Sterry W., Knorr F. (2009). Determination of the cuticula thickness of human and porcine hairs and their potential influence on the penetration of nanoparticles into the hair follicles. J. Biomed. Opt..

[126] Radtke M., Patzelt A., Knorr F., Lademann J., Netz R.R. (2017). Ratchet effect for nanoparticle transport in hair follicles. Eur. J. Pharm. Biopharm..

[127] Oaku Y., Shiroyama S., Otake H., Yajima Y., Abe A., Yamamoto N., Nagai N. (2024). Gum Arabic Enhances Hair Follicle-Targeting Drug Delivery of Minoxidil Nanocrystal Dispersions. Biol. Pharm. Bull..

[128] Ji Y., Li H., Li J., Yang G., Zhang W., Shen Y., Xu B., Liu J., Wen J., Song W. (2024). Hair Follicle-Targeted Delivery of Azelaic Acid Micro/Nanocrystals Promote the Treatment of Acne Vulgaris. Int. J. Nanomed..

[129] Lohan S.B., Saeidpour S., Colombo M., Staufenbiel S., Unbehauen M., Wolde-Kidan A., Netz R.R., Bodmeier R., Haag R., Teutloff C. (2020). Nanocrystals for Improved Drug Delivery of Dexamethasone in Skin Investigated by EPR Spectroscopy. Pharmaceutics.

[130] Klein A.L., Busch L., Lademann J., Meinke M.C., Keck C.M. (2025). Easy to use particle-mediated transport of various dissolved active agents into the hair follicles—A novel platform technology. Int. J. Pharm..

[131] Knorr F., Lademann J., Patzelt A., Sterry W., Blume-Peytavi U., Vogt A. (2009). Follicular transport route—Research progress and future perspectives. Eur. J. Pharm. Biopharm..

[132] Patzelt A., Lademann J. (2013). Drug delivery to hair follicles. Expert Opin. Drug Deliv..

[133] Blume-Peytavi U., Vogt A. (2011). Human hair follicle: Reservoir function and selective targeting. Br. J. Dermatol..

[134] Busch L., Asadzadeh D., Klein A.L., Suriyaamporn P., Vollrath M.K., Keck C.M., Meinke M.C. (2024). The penetration efficiency of a dissolved model drug into hair follicles depends on the concentration of added nanoparticles. Drug Deliv. Transl. Res..

[135] Yu Q., Wu X., Zhu Q., Wu W., Chen Z., Li Y., Lu Y. (2018). Enhanced transdermal delivery of meloxicam by nanocrystals: Preparation, in vitro and in vivo evaluation. Asian J. Pharm. Sci..

[136] Corrias F., Schlich M., Sinico C., Pireddu R., Valenti D., Fadda A.M., Marceddu S., Lai F. (2017). Nile red nanosuspensions as investigative model to study the follicular targeting of drug nanocrystals. Int. J. Pharm..

[137] Kumar M., Shanthi N., Mahato A.K., Soni S., Rajnikanth P.S. (2019). Preparation of luliconazole nanocrystals loaded hydrogel for improvement of dissolution and antifungal activity. Heliyon.

[138] Pelikh O., Keck C.M. (2020). Hair Follicle Targeting and Dermal Drug Delivery with Curcumin Drug Nanocrystals—Essential Influence of Excipients. Nanomaterials.

[139] Chaiprateep E.-O., Sengupta S., Keck C.M. (2025). Microneedle-Assisted Delivery of Curcumin: Evaluating the Effects of Needle Length and Formulation. Micromachines.

[140] Kobierski S., Ofori-Kwakye K., Müller R.H., Keck C.M. (2009). Resveratrol nanosuspensions for dermal application—Production, characterization, and physical stability. Pharmazie.

[141] Argenziano M., Ansari I.A., Muntoni E., Spagnolo R., Scomparin A., Cavalli R. (2022). Lipid-Coated Nanocrystals as a Tool for Improving the Antioxidant Activity of Resveratrol. Antioxidants.

[142] Karakucuk A., Tort S. (2020). Preparation, characterization and antimicrobial activity evaluation of electrospun PCL nanofiber composites of resveratrol nanocrystals. Pharm. Dev. Technol..

[143] Al Shaal L., Shegokar R., Müller R.H. (2011). Production and characterization of antioxidant apigenin nanocrystals as a novel UV skin protective formulation. Int. J. Pharm..

[144] Mitri K., Shegokar R., Gohla S., Anselmi C., Müller R.H. (2011). Lutein nanocrystals as antioxidant formulation for oral and dermal delivery. Int. J. Pharm..

[145] Li J., Ni W., Aisha M., Zhang J., Sun M. (2021). A rutin nanocrystal gel as an effective dermal delivery system for enhanced anti-photoaging application. Drug Dev. Ind. Pharm..

[146] Hassan A.S., Soliman G.M. (2022). Rutin Nanocrystals with Enhanced Anti-Inflammatory Activity: Preparation and Ex Vivo/In Vivo Evaluation in an Inflammatory Rat Model. Pharmaceutics.

[147] Stanisic D., Liu L.H.B., Dos Santos R.V., Costa A.F., Durán N., Tasic L. (2020). New Sustainable Process for Hesperidin Isolation and Anti-Ageing Effects of Hesperidin Nanocrystals. Molecules.

[148] Long J., Song J., Zhang X., Deng M., Xie L., Zhang L., Li X. (2020). Tea saponins as natural stabilizers for the production of hesperidin nanosuspensions. Int. J. Pharm..

[149] Manca M.L., Lai F., Pireddu R., Valenti D., Schlich M., Pini E., Ailuno G., Fadda A.M., Sinico C. (2020). Impact of nanosizing on dermal delivery and antioxidant activity of quercetin nanocrystals. J. Drug Deliv. Sci. Technol..

[150] Pant N., Wairkar S. (2022). Topical nanocrystals of bioflavonoids: A new technology platform for skin ailments. Int. J. Pharm..

[151] Rajasekar A., Devasena T. (2015). Facile Synthesis of Curcumin Nanocrystals and Validation of Its Antioxidant Activity Against Circulatory Toxicity in Wistar Rats. J. Nanosci. Nanotechnol..

[152] Xie L., Dai X., Li Y., Cao Y., Shi M., Li X. (2024). Pickering Emulsion of Curcumin Stabilized by Cellulose Nanocrystals/Chitosan Oligosaccharide: Effect in Promoting Wound Healing. Pharmaceutics.

[153] Salvioni L., Morelli L., Ochoa E., Labra M., Fiandra L., Palugan L., Prosperi D., Colombo M. (2021). The emerging role of nanotechnology in skincare. Adv. Colloid Interface Sci..

[154] Pireddu R., Caddeo C., Valenti D., Marongiu F., Scano A., Ennas G., Lai F., Fadda A.M., Sinico C. (2016). Diclofenac acid nanocrystals as an effective strategy to reduce in vivo skin inflammation by improving dermal drug bioavailability. Colloids Surf. B Biointerfaces.

[155] Ahmed I.S., Elnahas O.S., Assar N.H., Gad A.M., El Hosary R. (2020). Nanocrystals of Fusidic Acid for Dual Enhancement of Dermal Delivery and Antibacterial Activity: In Vitro, Ex Vivo and In Vivo Evaluation. Pharmaceutics.

[156] Rachmawati H., Al Shaal L., Müller R.H., Keck C.M. (2012). Development of curcumin nanocrystal: Physical aspects. J. Pharm. Sci..

[157] Nwozo O.S., Effiong E.M., Aja P.M., Awuchi C.G. (2023). Antioxidant, phytochemical, and therapeutic properties of medicinal plants: A review. Int. J. Food Prop..

[158] Rathinavel T., Ammashi S., Shanmugam G. (2021). Analgesic and anti-inflammatory potential of Lupeol isolated from Indian traditional medicinal plant Crateva adansonii screened through in vivo and in silico approaches. J. Genet. Eng. Biotechnol..

[159] Khumalo G.P., van Wyk B.E., Feng Y., Cock I.E. (2022). A review of the traditional use of southern African medicinal plants for the treatment of inflammation and inflammatory pain. J. Ethnopharmacol..

[160] Cedillo-Cortezano M., Martinez-Cuevas L.R., López J.A.M., Barrera López I.L., Escutia-Perez S., Petricevich V.L. (2024). Use of Medicinal Plants in the Process of Wound Healing: A Literature Review. Pharmaceuticals.

[161] Kulik-Siarek K., Klimek-Szczykutowicz M., Błońska-Sikora E., Zarembska E., Wrzosek M. (2025). Exploring the Antimicrobial Potential of Natural Substances and Their Applications in Cosmetic Formulations. Cosmetics.

[162] Aftab T., Hakeem K.R. (2021). Medicinal and Aromatic Plants.

[163] Bitwell C., Indra S.S., Luke C., Kakoma M.K. (2023). A review of modern and conventional extraction techniques and their applications for extracting phytochemicals from plants. Sci. Afr..

[164] Da Silva R.F., Carneiro C.N., de Sousa C.B.D.C., Gomez F.J., Espino M., Boiteux J., Fernández M.D.L.Á., Silva M.F., Dias F.D.S. (2022). Sustainable extraction bioactive compounds procedures in medicinal plants based on the principles of green analytical chemistry: A review. Microchem. J..

[165] Shah M., Murad W., Mubin S., Ullah O., Rehman N.U., Rahman M.H. (2022). Multiple health benefits of curcumin and its therapeutic potential. Environ. Sci. Pollut. Res. Int..

[166] Di Lorenzo R., Forgione F., Bernardi A., Sacchi A., Laneri S., Greco G. (2023). Clinical Studies on Topical Curcumin. Skin Pharmacol. Physiol..

[167] Kotian V., Koland M., Mutalik S. (2022). Nanocrystal-Based Topical Gels for Improving Wound Healing Efficacy of Curcumin. Crystals.

[168] Abraham A.M., Alnemari R.M., Jacob C., Keck C.M. (2020). PlantCrystals-Nanosized Plant Material for Improved Bioefficacy of Medical Plants. Materials.

[169] Knoth D., Alnemari R.M., Wiemann S., Keck C.M., Brüßler J. (2021). Fingerprint of Nature—Skin Penetration Analysis of a Stinging Nettle PlantCrystals Formulation. Cosmetics.

[170] Griffin S., Tittikpina N.K., Al-Marby A., Alkhayer R., Denezhkin P., Witek K., Gbogbo K.A., Batawila K., Duval R.E., Nasim M.J. (2016). Turning Waste into Value: Nanosized Natural Plant Materials of *Solanum incanum* L. and Pterocarpus erinaceus Poir with Promising Antimicrobial Activities. Pharmaceutics.

[171] Griffin S., Alkhayer R., Mirzoyan S., Turabyan A., Zucca P., Sarfraz M., Nasim M., Trchounian A., Rescigno A., Keck C. (2017). Nanosizing Cynomorium: Thumbs up for Potential Antifungal Applications. Inventions.

[172] Abraham A.M., Wiemann S., Ambreen G., Zhou J., Engelhardt K., Brüßler J., Bakowsky U., Li S.-M., Mandic R., Pocsfalvi G. (2022). Cucumber-Derived Exosome-like Vesicles and PlantCrystals for Improved Dermal Drug Delivery. Pharmaceutics.

[173] Abraham A.M., Alnemari R.M., Brüßler J., Keck C.M. (2021). Improved Antioxidant Capacity of Black Tea Waste Utilizing PlantCrystals. Molecules.

[174] Wang Y., Wei Y., Liao H., Fu H., Yang X., Xiang Q., Zhang S. (2023). Plant Exosome-like Nanoparticles as Biological Shuttles for Transdermal Drug Delivery. Bioengineering.

[175] Barzin M., Bagheri A.M., Ohadi M., Abhaji A.M., Salarpour S., Dehghannoudeh G. (2023). Application of plant-derived exosome-like nanoparticles in drug delivery. Pharm. Dev. Technol..

[176] Kürtösi B., Kazsoki A., Zelkó R. (2024). A Systematic Review on Plant-Derived Extracellular Vesicles as Drug Delivery Systems. Int. J. Mol. Sci..

[177] Langellotto M.D., Rassu G., Serri C., Demartis S., Giunchedi P., Gavini E. (2025). Plant-derived extracellular vesicles: A synergetic combination of a drug delivery system and a source of natural bioactive compounds. Drug Deliv. Transl. Res..

[178] Alkhaldi M., Sehra T., Sengupta S., Keck C.M. (2024). Extracellular Vesicles and PlantCrystals for Improved Bioavailability of Curcumin as a BCS Class IV Drug. Molecules.

[179] Sehra T., Alkhaldi M., Sengupta S., Keck C.M. (2025). Milk thistle (*Silybum marianum*) based Exosomes-like vesicles and PlantCrystals for improved bioavailability of active compounds. Drug Deliv. Transl. Res..

